# Natural hybridization and genetic and morphological variation between two epiphytic bromeliads

**DOI:** 10.1093/aobpla/plx061

**Published:** 2017-11-25

**Authors:** Jordana Neri, Tânia Wendt, Clarisse Palma-Silva

**Affiliations:** Programa de Pós Graduação em Botânica, Departamento de Botânica, Museu Nacional, Universidade Federal do Rio de Janeiro, Quinta da Boa Vista, São Cristóvão, Rio de Janeiro, RJ, Brazil; Departamento de Botânica, Instituto de Biologia, Universidade Federal do Rio de Janeiro, Rio de Janeiro, RJ, Brazil; Programa de Pós Graduação em Ecologia, Departamento de Ecologia – Universidade Estadual Paulista Julio Mesquita Filho, Rio Claro, SP, Brazil

**Keywords:** Atlantic Forest, Bromeliaceae, floral traits, hybrids, reproductive barriers, species integrity, sympatric

## Abstract

Reproductive isolation is of fundamental importance for maintaining species boundaries in sympatry. Here, we examine the genetic and morphological differences between two closely related bromeliad species: *Vriesea simplex* and *Vriesea scalaris*. Furthermore, we examined the occurrence of natural hybridization and discuss the action of reproductive isolation barriers. Nuclear genomic admixture suggests hybridization in sympatric populations, although interspecific gene flow is low among species in all sympatric zones (*N*_e_*m* < 0.5). Thus, morphological and genetic divergence (10.99 %) between species can be maintained despite ongoing natural hybridization. Cross-evaluation of our genetic and morphological data suggests that species integrity is maintained by the simultaneous action of multiple barriers, such as divergent reproductive systems among species, differences in floral traits and low hybrid seed viability.

## Teaser

We investigated the potential evolutionary mechanisms associated with maintenance of reproductive species barriers between V. simplex and V. scalaris through morphological and genetic data. Nuclear genomic admixture suggests hybridization in sympatric populations, although interspecific gene flow is low among species in all sympatric zones (N < 0.5). Thus, morphological and genetic divergence (10.99%) between species can be maintained despite ongoing natural hybridization. Cross-evaluation of our genetic and morphological data suggests that species integrity is maintained by the simultaneous action of multiple barriers, such as divergent reproductive systems among species and differences in floral traits.

## Introduction

Natural hybridization is an important process in plant evolution. It has been estimated that 30–70 % of all flowering plant species have hybridization events in their phylogenetic histories (Ehrlich and Wilson 1991; Rieseberg 1995; Soltis and Soltis 2009). Therefore, hybrid zones are interesting models for studying the evolution of reproductive barriers, the role of selection in maintaining species differences and how phenotypic traits differ between hybridizing populations (Abbott *et al.* 2013; Arnold 2014).

The degree of the reproductive isolation barrier among related species is an important factor that influences the genetic integrity of a species and the probability of forming a hybrid (Coyne and Orr 2004). One of the key points in evolutionary biology is to determine how reproductive barriers limit introgressive gene flow and hybridization (Coyne and Orr 2004). Congruent hybridization patterns can either unify populations of species or preserve two populations of species with different allele frequencies (Servedio and Kirkpatrick 1997). Asymmetric gene flow is important to understanding the factors that determine the maintenance of species identity. Some of these underlying factors include variations in the reproductive system (Hu 2015) and flower size (floral traits). Flower size may also be a major structural barrier to hybridization that is generally asymmetrical (Williams and Rouse 1988). This feature is particularly important because flower sizes generally differ between hybridizing species (Field *et al.* 2011).

Reproductive isolation barriers can function prior to mating (premating), after mating but before zygote formation (postmating–prezygotic) and after zygote formation (postzygotic) (Ramsey *et al.* 2003; Lowry *et al.* 2008; Widmer *et al.* 2009; Baack *et al.* 2015). The identification of these different barriers in studies of reproductive isolation requires the integration of several approaches, such as morphological analysis, population genetics, cytogenetics, and genome size and manual reproductive experiments, which can detect mechanisms that influence interspecific gene flow (Wendt *et al.* 2001, 2002; Moccia *et al.* 2007; Pinheiro *et al.* 2010; Palma-Silva *et al.* 2011; Brys *et al.* 2013; Briscoe Runquist *et al.* 2014; Marques *et al.* 2014; Pinheiro *et al.* 2015; Twyford *et al.* 2015).

Mating systems are recognized as key barriers to reproductive isolation (Jain 1976; Coyne and Orr 2004), although their role in speciation and species cohesion is not completely understood, especially for plants with mixed mating systems (Hu 2015). The floral adaptations that allow autonomous selfing are assumed to offer effective mechanical protection against heterospecific mating and thus to contribute to reproductive isolation (Wright *et al.* 2013). Empirical support for selfing as a reproductive barrier was reported for different plant groups (Fishman and Wyatt 1999; Wendt *et al.* 2002; Fishman and Stratton 2004; Lowe and Abbott 2004; Martin and Willis 2007; Matallana *et al.* 2010). In addition, in sympatric species with a shared generalized floral morphology and pollinator community, intense competition for pollination and fitness costs related to hybridization may select for floral traits that contribute to prezygotic isolation (Servedio and Noor 2003). For example, Beans (2014) suggests that the divergence of floral traits in sympatric and allopatric populations may evolve in response to competition for pollinator resources or in response to costs associated with sharing pollinators with other species.

Neotropical plant radiations provide a perfect system for examining potential gene flow among closely related species. The Bromeliaceae, in particular, have been used as a model group in studies of evolution of reproductive isolation and speciation in Neotropical regions (Schulte *et al.* 2010; Palma-Silva *et al.* 2011, 2015, 2016a, b; Wagner *et al.* 2015; Lexer *et al.* 2016; Zanella *et al.* 2016), mainly due to its high diversity and recent adaptive radiation (Benzing 2000; Givnish *et al.* 2011, 2014).

Here we investigated natural hybridization and the factors involved in the maintenance of phenotypic and genetic differentiation between two recently diverged species (Barfuss *et al.* 2016) that occur in the Brazilian Atlantic Forest: *Vriesea simplex* and *Vriesea scalaris*. The two species are self-compatible (Matallana *et al.* 2010), but their reproductive systems are divergent, ranging from predominantly outcrossing and pollinator dependent in *V. simplex* to highly selfing in *V. scalaris* (Neri *et al.* 2017). Both species exhibit similar pollination syndromes, their flowers are visited by hummingbirds (*Phaethornis eurynome* and *Ramphodon naevious*) and overlap in flowering time occurs in sympatry (Wendt *et al.* 2008). In addition, artificial hybrids (F_1_) were obtained via manual crossing experiments involving these two taxa (Neri *et al.* 2017), confirming interspecific compatibility. Despite reports of artificial cross-compatibilities and observation of putative hybrids in the field, the extent of gene exchange between these two species in natural populations has not been investigated.

We investigated four allopatric and three sympatric populations using a combined set of microsatellite markers and multivariate analyses of morphology to answer the following questions: (i) How do genetic and morphological differences between sympatric and allopatric populations contribute to reproductive isolation between *V. simplex* and *V. scalaris*? (ii) Do *V. simplex* and *V. scalaris* hybridize in the wild, as suggested by artificial crosses and observations of putative hybrids in the field? If yes, are the patterns of hybridization and gene flow (migration) similar or asymmetrical across sympatric populations? (iii) What is the importance of the action of different prezygotic and postzygotic barriers in maintaining species integrity?

## Methods

### Population samples and DNA extraction

**Figure 1.**
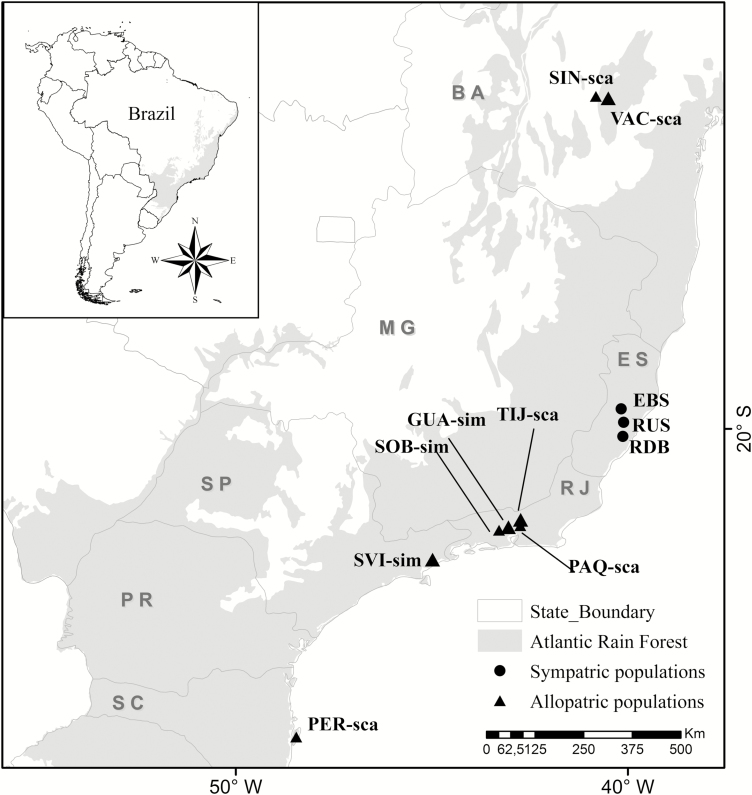
Map of localities of samples collected from sympatric and allopatric populations of *Vriesea simplex* and *V. scalaris* in the Atlantic rainforest used for study of hybridization and morphometric analysis. *Vriesea simplex*, ‘sim’; *V. scalaris*, ‘sca’. For abbreviations of populations, see Table 1.

*Vriesea simplex* and *V. scalaris* are epiphytic species that occur in mesophilic environments and well-preserved habitats with high humidity in the Brazilian Atlantic rainforest (Fig. 1). *Vriesea simplex* has a narrow distribution (Bahia, Espírito Santo, Rio de Janeiro and São Paulo), whereas *V. scalaris* has a widespread distribution (Pernambuco to Rio Grande do Sul states; Forzza *et al.* 2015). We sampled three hybrid zones: Santa Lucia (EBS; 108 individuals), Ruschi (RUS; 40 individuals) and Duas Bocas (RDB; 33 individuals); two allopatric collection locales of *V. simplex*: Guapimirim (GUA; 16 individuals) and Soberbo (SOB; 18 individuals); and two allopatric collection locales of *V. scalaris*: Peri (PER; 20 individuals) and Sincorá (SIN; 10 individuals). In total, 255 flowering or fruiting plants were sampled (Table 1; Fig. 1). Voucher information for each collection locale and species is given in **Supporting Information—Table S1**. The identified hybrids and their intermediate morphology are described in **Supporting Information—Table S2**. Fresh leaves from each individual were collected and stored in silica gel. Total genomic DNA was extracted using a Invisorb Spin Plant Mini Kit (Stratec Biomedical AG, Birkenfeld, Germany) according to the manufacturer’s instructions.

**Table 1.** Allopatric and sympatric populations of *Vriesea simplex* and *V. scalaris* sampled in this study. Morphometric (*N*): number of individuals sampled for morphometric analysis; SSR (*N*): number of individuals sampled for genetic analysis.

Species	Collection locales	Code	State	City	Morphometric (N)	SSR (N)	Long	Lat
*V. scalaris*	Vale do Capão	VAC	Bahia	Palmeiras	03	–	−41.477222	−12.5425
*V. scalaris*	Sincorá	SIN	Bahia	Igatú	03	10	−41.574167	−12.511389
*V. simplex /V. scalaris* and hybrids	Duas Bocas	RDB	Espírito Santo	Cariacica	01/10	16/15/2	−40.532711	−20.298181
*V. simplex* /*V. scalaris* and hybrids	Santa Lucia	EBS	Espírito Santo	Santa Teresa	20/15	57/39/12	−40.529539	−19.972567
*V. simplex*/*V. scalaris* and hybrids	Ruschi	RUS	Espírito Santo	Santa Teresa	02/06	18/14/8	−40.563903	−19.906006
*V. simplex*	Guapimirim	GUA	Rio de Janeiro	Guapimirim	12	16	−42.984789	−22.482239
*V. scalaris*	Tijuca	TIJ	Rio de Janeiro	Rio de Janeiro	16	–	−43.281178	−22.822156
*V. scalaris*	Paquequer	PAQ	Rio de Janeiro	Teresópolis	07	–	−42.981883	−22.437581
*V. simplex*	Soberbo	SOB	Rio de Janeiro	Teresópolis	20	28	−42.983478	−22.473631
*V. simplex*	Santa Virgínia	SVI	São Paulo	São Luiz do Paraitinga	05	–	−45.147028	−23.337611
*V. scalaris*	Peri	PER	Santa Catarina	Florianópolis	19	20	−48.527786	−27.753942

### Nuclear microsatellite markers and genotyping

To study the patterns of genetic diversity and genomic admixture in sympatric and allopatric populations, we used 15 nuclear microsatellite loci (SSR) previously developed for other bromeliad species. Six loci were isolated from *V*. *simplex* (Vs1, Vs2, Vs8, Vs9, Vs10 and Vs19; Neri *et al.* 2015), six loci from *V*. *gigantea* (VgB01, VgB10, VgB12, VgG02, VgG04 and VgG05; Palma-Silva *et al.* 2007), two loci from *Tillandsia fasciculata* (E6 and E6b; Boneh *et al.* 2003) and one locus from *Aechmea caudata* (Ac01; Goetze *et al.* 2013). For each SSR, the forward primers were synthesized with an M13 tail (5′-CACGACGTTGTAAAACGAC-3′) to allow for marking and multiplexing fluorescent dyes during the amplification and genotyping procedures. All PCR amplification reactions were performed in a Thermal Cycler (Applied Biosystems, Foster City, CA, USA) following the protocol described by Palma-Silva *et al.* (2007). The microsatellite alleles were resolved on an 3500 DNA Analyzer automated sequencer (Applied Biosystems) and sized against the GeneScan 500 LIZ molecular size standard (Applied Biosystems) using GENEMARKER Demo version 1.97 software (SoftGenetics, State College, PA, USA). Microchecker software (Van Oosterhout *et al.* 2004) was used to check for null alleles.

### Statistical analysis

#### Patterns of genomic admixture for hybrid detection.

Bayesian analysis was performed in STRUCTURE 2.3.2 software (Pritchard *et al.* 2000) and carried out under the admixture model assuming independent allele frequencies, using a burn-in period of 100000, run length of 500000 and 10 replicates per *K* ranging from 1 to 10 with all populations in the data set. Using the method proposed by Evanno *et al.* (2005), which is based on an *ad hoc* measure of Δ*K*, we determined the highest number of clusters (*K*) as *K* = 2, corresponding to the two species. We used the *K* = 2 model and 10 replicates per *K* because we assumed that the two species contributed to the gene pool of the sample. Allopatric populations of each species were used as reference samples of pure *V. simplex* and *V. scalaris.* Follow-up analyses were performed separately for each hybrid zone, in each case including the specimens from the allopatric populations as reference samples for each species. We investigated the genetic structural patterns of each hybrid zone separately because the allelic frequencies within the populations of each species were different (see Table 3), particularly because of the high and variable selfing rates observed for *V. scalaris* (Table 3). It is also important to analyse hybrid zones separately when gene flow is restricted among populations, leading to high divergence and different genomic architecture among hybrid zones (Pinheiro *et al.* 2010; Marques *et al.* 2014; Twyford *et al.* 2015; Zanella *et al.* 2016). Following the procedure described by Burgarella *et al.* (2009), STRUCTURE was used to classify individuals among the two parental species and hybrids, using a threshold of *q* ≥ 0.90 to classify pure individuals of *V. scalaris*, *q* ≤ 0.10 to classify pure individuals of *V. simplex* and 0.10 < *q* < 0.90 to classify hybrid individuals (Vähä and Primmer 2006).

In addition, the clustering method of Anderson and Thompson (2002) implemented in the NEWHYBRIDS version 1.1 software was used to test assignment of individuals into different genotypic classes (pure parental species 1 or 2, F_1_, F_2_ and backcross), using a threshold value of *q* = 0.75; individuals with *q* < 0.75 remained unassigned.

#### Genetic diversity.

Populations and loci were characterized for *V. simplex*, *V. scalaris* and their hybrids based on number of alleles, allelic richness, variance in allele size, observed and expected heterozygosity and inbreeding coefficient (*F*_IS_; Weir and Cockerham 1984) using the MSA (Dieringer and Schlötterer 2003) and FSTAT softwares (Goudet 1995). We also estimated the private alleles of each species and hybrids using GenAlEx software (Peakall and Smouse 2006).

Departures from Hardy–Weinberg equilibrium (HWE) were tested using the web-based GENEPOP 3.5 software (Raymond and Rousset 1995). Using the *F*_IS_ we calculated the apparent outcrossing rate (*t*_a_), according to the following formula: *t*_a_ = (1 − *F*_IS_)/(1 + *F*_IS_) (Goodwillie *et al.* 2005). We assume that predominantly selfing populations have *t*_a_ ≤ 0.2, mixed systems have 0.2 < *t*_a_ ≤ 0.8 and predominantly outcrossing populations have *t*_a_ > 0.8 (Goodwillie *et al.* 2005).

Partitioning of genetic diversity within and among *V. simplex* and *V. scalaris* groups was evaluated by an analysis of molecular variance (AMOVA) implemented in the software ARLEQUIN 3.1 (Excoffier *et al.* 2005). Principle coordinate analysis (PCoA) was used on the entire data set to visualize genetic differences between species and allopatric and sympatric populations, and to examine the genetic status of plants along the contact zone, implemented in the GenAlEx program (Peakall and Smouse 2006).

#### Effective population size and migration rate.

We calculated the effective population size (*N*_e_) of *V. simplex* and *V. scalaris* because we expected that introgression might occur more intensely in populations with a smaller effective population size. Effective migration rates (*N*_e_*m*) were calculated to identify the direction of the gene flow between species (introgression). Theta (4*N*_e_µ = and µ = mutation rate) and effective migration rates (*N*_e_*m*) were estimated between pairs of sympatric populations of *V. simplex* and *V. scalaris* following a coalescent theory and maximum-likelihood-based approach using MIGRATE 3.0.3 software (Beerli and Felsenstein 1999). The computations were carried out under the infinite allele model (Kimura and Crow 1964). Effective population size values were estimated from theta values by assuming a microsatellite µ rate of 10^−3^ per gamete per generation (Zhang and Hewitt 2003).

#### Sampling and morphometric analysis.

Morphometric analyses of vegetative and reproductive traits of pure species (individuals of both species) and hybrids were performed in two sympatric populations, three allopatric collection locales of *V. simplex* and five allopatric collection locales of *V. scalaris* (Table 1; Fig. 1). All individuals included in morphometric analyses were identified as parental species or hybrids using SSR genotypes based on STRUCTURE analysis, as described above.

Individuals were collected and measured based on availability and accessibility. In total, we measured 139 flowering specimens from both parental species and hybrids (Table 1). Twenty-four quantitative traits (6 vegetative and 18 reproductive; Table 5) were measured using callipers. The flowers were collected and preserved in 70 % ethanol. A discriminant analysis (canonical variance analysis; CVA) was performed in the STATISTICA8 program for Windows 4.2 (StatSoft 1993) to test the partition among predefined clusters (*V. simplex* allopatric, *V. scalaris* allopatric, *V. simplex* sympatric, *V. scalaris* sympatric and hybrids) and to identify traits that contribute most to species discrimination.

We used the most significant traits for species discrimination in CVA to examine the extent of variation among sympatric and allopatric collection locales between species. These comparisons were performed using analysis of variance (ANOVA), followed by Tukey’s test through the general linear model. These statistical analyses were performed using R software (Core Team 2015).

## Results

### Genetic composition of hybrid zones

**Figure 2.**
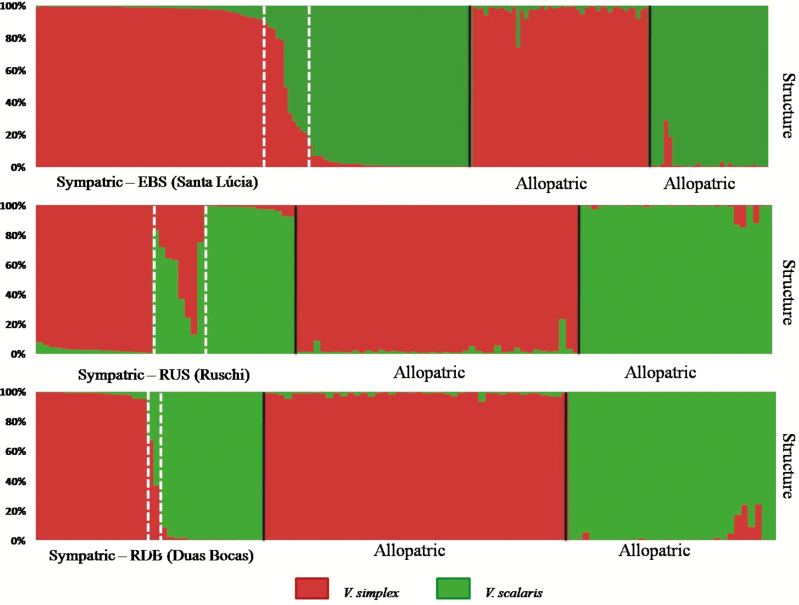
Bayesian admixture proportions (*Q*) of each *Vriesea simplex* and *V. scalaris* individual estimated in STRUCTURE, assuming *K* = 2, for each hybrid zone (sympatric populations) and allopatric population. Red colour indicates pure individuals of *V. simplex* and green colour indicates pure individuals of *V. scalaris*. Dashed white line indicates the hybrid.

**Figure 3.**
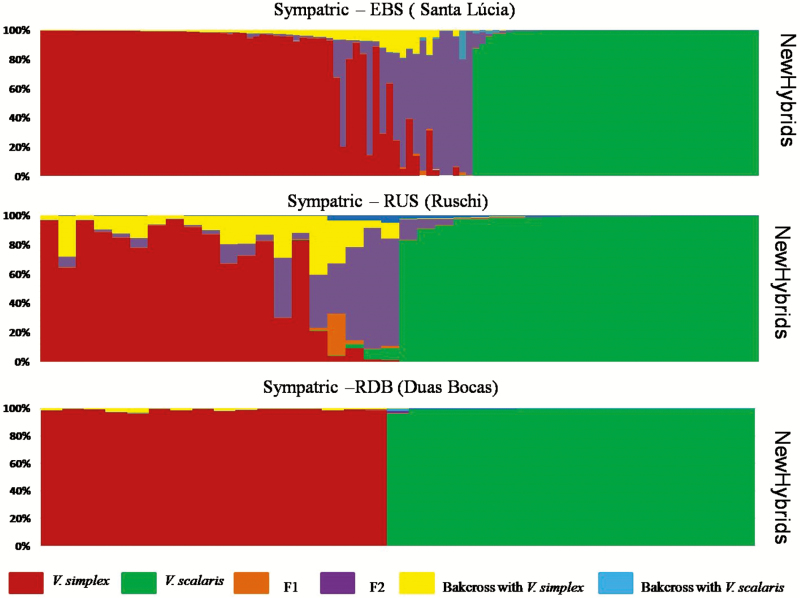
Bayesian admixture proportions (*Q*) of each *Vriesea simplex* and *V. scalaris* individual estimated in NEWHYBRIDS for each sympatric population. The proportion of colour in each bar represents an individual’s assignment probability based on different categories (pure parental species, hybrid F_1_, F_2_ and backcrosses).

Genomic admixture analysis with Bayesian STRUCTURE results for sympatric populations indicated hybridization between *V. simplex* and *V. scalaris*, with a total of 22 hybrids identified among 252 individuals sampled (12 % of the total individuals sampled in sympatric populations; threshold: 0.10 < *q* < 0.90; Fig. 2). The EBS population had 12 hybrids among 108 individuals, the RUS population had 8 hybrids among 40 individuals and the RDB population had only 2 hybrids among 33 individuals. Most hybrids identified in STRUCTURE were not assigned into any hybrid class using NEWHYBRIDS. In total, NEWHYBRIDS was able to classify 10 hybrid individuals, all as F_2_, 8 in the EBS population, and 2 in the RUS population. In the RDB population, no hybrid was classified by NEWHYBRIDS (Fig. 3).

### Nuclear microsatellite diversity

Levels of genetic diversity differed strongly between species (Table 2), with *V*. *simplex* having a total of 262 alleles (ranging from 6 to 40 alleles per locus) and *V. scalaris* having a total of 92 alleles (ranging from 3 to 18 alleles per locus). Hybrids presented a total of 110 alleles (ranging from 3 to 18 alleles per locus) (Table 2). The mean observed and expected heterozygosities per locus were 0.550 and 0.684, respectively, for *V. simplex*, 0.170 and 0.347, respectively, for *V. scalaris*, and 0.301 and 0.487, respectively, for hybrids (Table 2). The *F*_IS_ was high and departed significantly from HWE in almost all loci (Table 2) for both species and hybrids. *F*_IS_ values were consistently lower in *V. simplex* than in *V. scalaris* (Table 3). We observed a higher number of private alleles for *V. simplex*, with 128 private alleles (out of 262 alleles), than in *V. scalaris*, with 6 private alleles (out of 92 alleles), or in hybrids, with 7 private alleles (out of 110 alleles).

**Table 2.** Genetic variability at 15 nuclear microsatellite loci in *Vriesea simplex*, *Vriesea scalaris,* and their hybrids, including locus name, number of alleles (*A*), allelic richness (AR), observed *(H*_O_) and expected (*H*_E_) heterozygosity, and inbreeding coefficient (*F*_IS_) for each locus. ^NA^ departed from HWE cannot be calculated. Inbreeding coefficient (F_IS_) departed significantly from HWE are indicated by asterisks (*P < 0.05, **P < 0.0001).

	*Vriesea simplex* (*N* = 135)					Hybrids (*N* = 22)					*Vriesea scalaris* (*N* = 98)				
Locus	*A*	AR	*H*_O_	*H*_E_	*F*_IS_	*A*	AR	*H*_O_	*H*_E_	*F*_IS_	*A*	AR	*H*_O_	*H*_E_	*F*_IS_
Vs1	14	6.60	0.500	0.650	0.295**	11	3.31	0.319	0.657	0.408*	10	6.69	0.235	0.607	0.607**
Vs2	23	7.61	0.529	0.661	0.097*	6	2.38	0.458	0.697	0.219	4	3.06	0.176	0.354	0.479**
Vs8	11	4.09	0.199	0.354	0.341**	3	1.19	0.075	0.081	−0.064	4	2.25	0.061	0.149	0.570*
Vs9	40	14.80	0.796	0.963	0.169*	14	3.49	0.333	0.687	0.401*	11	6.45	0.087	0.437	0.816**
Vs10	14	5.66	0.495	0.508	−0.089	5	2.02	0.543	0.504	−0.215	4	2.18	0.402	0.258	−0.575*
Vs19	35	14.65	0.797	0.944	0.151**	13	2.89	0.236	0.619	0.529*	9	6.30	0.060	0.456	0.884**
Ac01	8	5.08	0.361	0.569	0.262**	6	2.02	0.131	0.314	0.484	1	1.00	0.000	0.000	1.000^NA^
e6	12	5.98	0.694	0.676	−0.015	4	1.86	0.265	0.332	0.067	7	3.63	0.461	0.498	0.058**
e6b	10	6.23	0.707	0.749	0.067	5	1.83	0.248	0.265	−0.090	7	4.10	0.134	0.383	0.659**
VgB01	7	4.73	0.287	0.600	0.440**	4	1.93	0.123	0.379	0.593*	3	2.13	0.148	0.173	0.115
VgB10	34	14.07	0.743	0.918	0.199**	18	3.71	0.607	0.845	0.149*	18	9.24	0.172	0.669	0.752**
VgB12	6	3.29	0.374	0.456	0.063	4	2.07	0.383	0.579	0.214	2	1.96	0.126	0.216	0.419
VgG02	20	6.66	0.598	0.690	0.162**	5	1.61	0.153	0.211	0.147	2	1.57	0.000	0.056	1.000*
VgG05	14	6.69	0.667	0.791	0.174*	7	2.81	0.288	0.520	0.331*	6	4.01	0.053	0.342	0.874**
VgA04	14	6.35	0.503	0.734	0.202**	5	1.72	0.357	0.618	0.181	4	3.04	0.089	0.255	0.631**
Overall/ mean	262	7.04	0.550	0.684	0.168	110	2.32	0.301	0.487	0.224	92	3.84	0.170	0.347	0.521

**Table 3.** Characterization of populations of *Vriesea simplex*, *V. scalaris* and their hybrids, with 15 nuclear microsatellite markers, including the number of individuals sampled (*N*), number of alleles (*A*), number of private alleles (Ap), allelic richness (AR), variance in allele size (Var), observed (*H*_O_) and expected (*H*_E_) heterozygosity, inbreeding coefficient (*F*_IS_), and apparent outcrossing rate (*t*_a_) for each population. Inbreeding coefficient (*F*_IS_) departed significantly from HWE are indicated by asterisks (*P < 0.05).

Species (samples size)	*N*	*A*	Ap	AR	Var	*H*_ O _	*H*_ E _	*F*_ IS _	*t*_ a _
Allopatric—*V. simplex*									
Guapimirim, RJ	16	111	18	6.75	33.67	0.545	0.668	0.196*	0.693
Soberbo, RJ	28	157	32	7.80	37.14	0.584	0.732	0.215*	0.658
Allopatric—*V. scalaris*									
Peri, SC	20	29	4	1.88	17.19	0.195	0.221	0.145*	0.753
Sincorá, BA	10	33	7	2.20	17.34	0.134	0.252	0.509*	0.408
Simpatric—Santa Lucia, ES									
*V. simplex*	57	170	46	6.50	27.59	0.556	0.649	0.178*	0.704
Hybrid	12	83	50	2.33	29.09	0.411	0.594	0.318*	0.592
*V. scalaris*	39	55	19	2.73	36.55	0.105	0.312	0.673*	0.158
Simpatric—Ruschi, ES									
*V. simplex*	18	102	9	6.11	42.04	0.538	0.649	0.204*	0.691
Hybrid	8	64	20	2.26	33.14	0.359	0.557	0.371*	0.653
*V. scalaris*	14	42	8	2.69	16.26	0.084	0.385	0.797*	0.225
Simpatric—Rebio Duas Bocas, ES									
*V. simplex*	16	86	6	5.71	23.66	0.545	0.616	0.102*	0.845
Hybrid	2	20	6	1.33	28.85	0.411	0.594	0.200*	1.000
*V. scalaris*	15	34	11	2.16	19.90	0.187	0.359	0.435*	0.358

The number of alleles in populations ranged from 102 to 170 in *V. simplex* and from 29 to 42 in *V. scalaris* (Table 3). Population-level *F*_IS_ values departed significantly from HWE in almost all populations. The *F*_IS_ values were higher with significant heterozygote deficits more prevalent in *V. scalaris* (*F*_IS_ = 0.511) than in *V. simplex* (*F*_IS_ = 0.179), consistent with differences in their reproductive systems (Neri *et al.* 2017). In agreement with mating system variation between species, the apparent outcrossing rates (*t*_a_) were higher for *V. simplex*, ranging from 0.658 to 0.845, than for *V. scalaris*, ranging from 0.158 to 0.753 (Table 3). Despite this, all *t*_a_ values for both species were between 0.2 and 0.8, suggesting mixed systems for both species (Table 3). In addition, *V. simplex t*_a_ values were similar among sympatric and allopatric populations, but *V. scalaris* sympatric populations had lower *t*_a_ values than allopatric populations, indicating selfing rates may be higher in all sympatric populations (Table 3). Hybrids showed, on average, an intermediate genetic diversity index compared to purebred species.

### Genetic differentiation and nuclear migration rates between species

**Figure 4.**
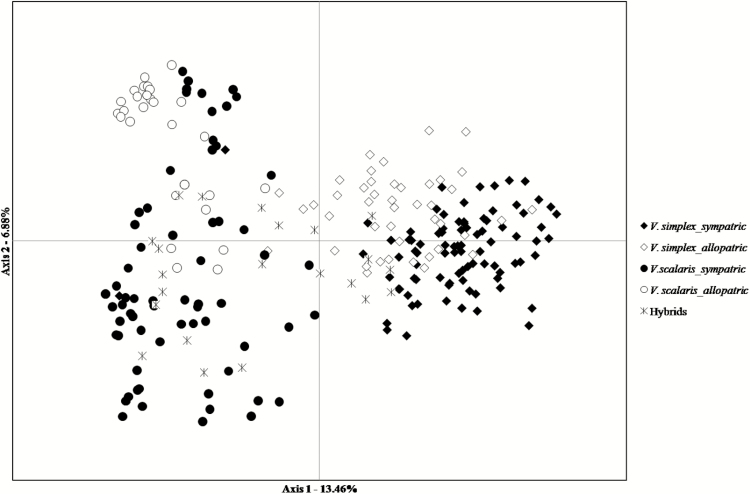
Principal coordinate analysis of 15 nuSSR data for *Vriesea simplex*, *V. scalaris* and hybrids. Axis 1 and axis 2 account for 33.1 and 16.9 % of the variance, respectively.

AMOVA results showed that genomic differentiation between species was low (10.99 %), but still highly significant (*P* < 0.001; Table 4). The separated AMOVA model for each species indicated lower genetic structure among populations of *V. simplex* (*F*_ST_ = 0.069; *P* < 0.001) than for *V. scalaris* (*F*_ST_ = 0.416; *P* < 0.001) (Table 4). The PCoA produced two defined groups of pure *V. simplex* and pure *V. scalaris*. The hybrids did not form an intermediate group and several hybrids were grouped with *V. scalaris* (Fig. 4).

**Table 4.** AMOVA for 15 nuclear microsatellites with two hierarchical levels, including *Vriesea simplex* and *V. scalaris* pure individuals in sympatric and allopatric populations.

	Source of variation	**Variation %**	***F*-statistics**	*P*-value
By species	Among species	10.99	*F*_CT_ =0.10999	<0.001
	Among population within species	16.86	*F*_SC_ =0.19086	<0.001
	Within populations	74.01	*F*_ST_ =0.27986	<0.001
*Vriesea simplex*				
	Among populations	6.98	*F*_ST_ =0.069	<0.001
	Within populations	93.01		<0.001
*Vriesea scalaris*				
	Among populations	41.67	*F*_ST_ =0.416	<0.001
	Within populations	58.32		<0.001

**Figure 5.**
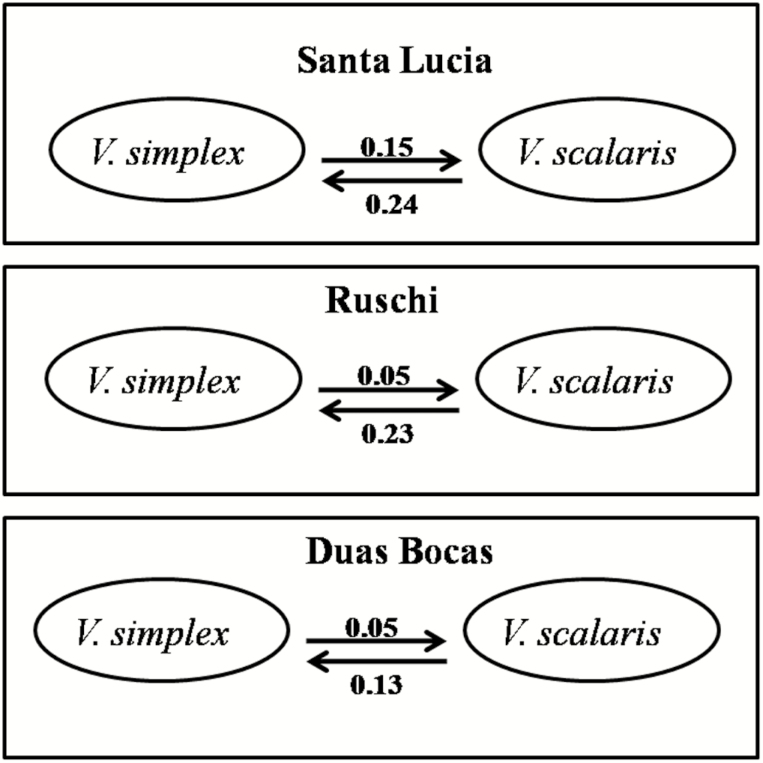
Bidirectional migration rates (effective number of migrants, *N*_e_*m*) in three sympatric populations of *Vriesea simplex* and *V*. *scalaris*.

The maximum-likelihood-based estimates of the effective numbers of migrants (*N*_e_*m*) for sympatric populations of *V. simplex* and *V. scalaris* were very low among species, suggesting restricted interspecific gene flow. Although low, interspecific migration rates were asymmetric towards *V. simplex*, with larger *N*_e_*m* values from *V. scalaris* into *V. simplex* (Fig. 5). The *N*_e_ sizes were larger for *V. simplex* (ST, *N*_e_ = 3080; RUS, *N*_e_ = 5972.50; RBD, *N*_e_ = 5362.50; GUA, *N*_e_ = 3.60; and SOB, *N*_e_ = 4.21) than for *V. scalaris* (ST, *N*_e_ = 1041.55; RUS, *N*_e_ = 395.05; RDB, *N*_e_ = 404.97; SIN, *N*_e_ = 426.95; and PER, *N*_e_ = 115.65).

### Morphometric analysis of vegetative and reproductive traits among species

**Figure 6.**
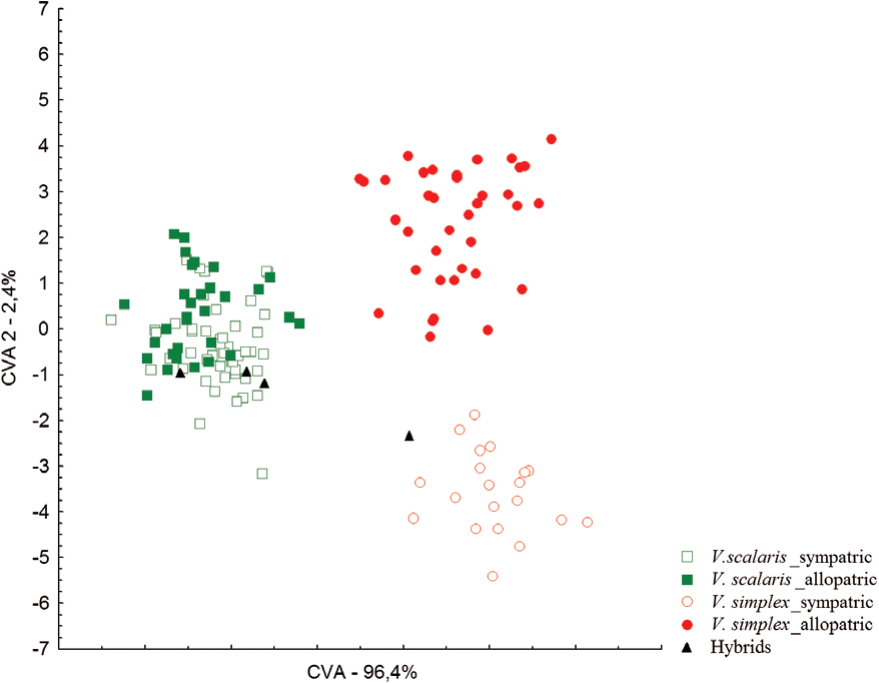
Scatter plot of the scores derived from discriminant functions CVA1 versus CVA2 produced by stepwise discriminate analysis (CVA) applied to 24 morphometric characters for *Vriesea simplex* and *V. scalaris.*

**Figure 7.**
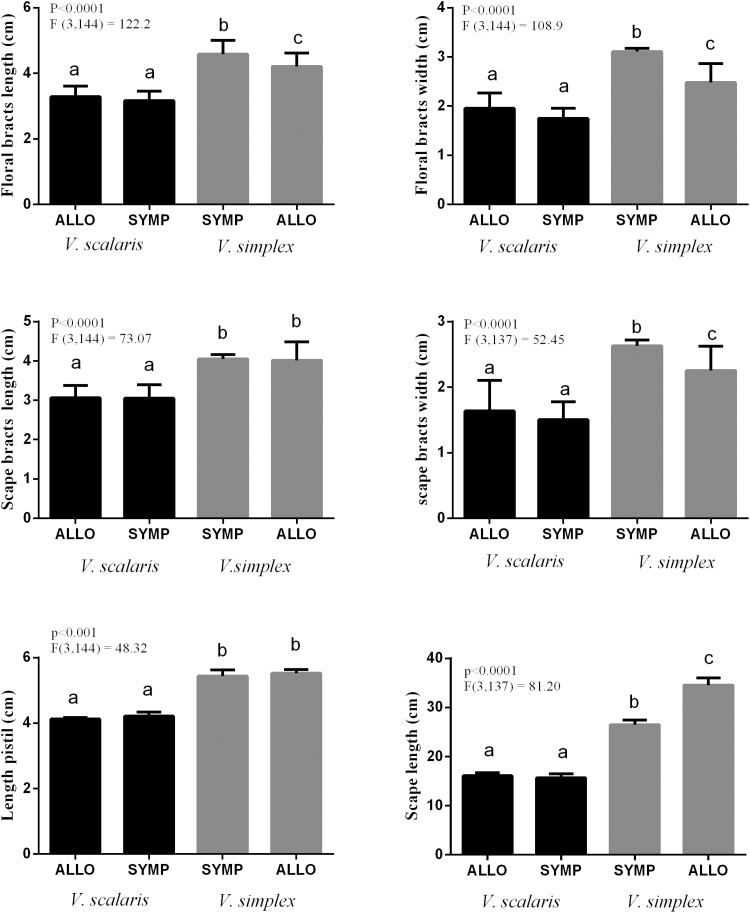
Comparison of six characters between sympatric and allopatric populations of *Vriesea simplex* and *V. scalaris* through ANOVA. Six allopatric populations of *V. scalaris*, four allopatric populations of *V. simplex* and three sympatric populations were sampled. Means ± SE followed by superscript letters are significantly different (*P* < 0.05, Tukey’s test).

CVA morphometric analysis of 24 characters (18 reproductive and 6 vegetative) distinguished two well-defined groups, consistent with parental species identified using microsatellites. Hybrids did not present intermediate trait grouping with one or the other species (Fig. 6). The first CVA axis accumulated 97 % of the total variation among clusters (Fig. 6). The six variables that contributed most to this axis were floral traits: scape length; length and width of the floral bract; length and width of the bract floral scape; and pistil length (Table 5). Additionally, three out of the six floral traits (floral bract length, floral bract width and scape bract width) were significantly higher in sympatric populations than in allopatric populations of *V. simplex* (Fig. 7). Scape length was significantly lower in sympatric than in allopatric populations of *V. simplex* (Fig. 7).

**Table 5.** Standardized coefficients for canonical variables derived from discriminant function analysis (CVA) from 11 populations of *Vriesea simplex* and *V. scalaris* along the Atlantic rainforest, Brazil. The sublimated characters are those that contribute the most to the species separation according to the values of CVA1 and CVA2.

Characters	CVA1	CVA2
*Scape length*	0.407671	0.420156
*Floral bract width*	0.540610	−0.063002
*Floral bract length*	0.490502	−0.293909
*Scape bracts width*	0.416668	0.118613
*Scape bracts length*	0.362053	−0.092482
*Length pistil*	0.332938	0.143569
Leaf heath length	0.012159	0.050331
Leaf heath width	0.177408	0.320468
Leaf blade length	0.20957	0.610379
Leaf blade width	0.203816	0.071918
Inflorescence total length	0.275906	0.382661
Rachis length	0.125729	0.276047
Flowers number	0.153994	0.101936
Sepal width	0.128110	0.003864
Sepal length	0.041543	0.062018
Petal width	0.290863	−0.054088
Petal length	0.005314	0.042810
Anther length	0.077106	−0.028683
Pedicle length	0.224256	0.022448
Anther–stigma distance	0.164429	0.026982
Stamen length	0.294832	0.056851
Fillet length	0.255827	0.090300
Rosette diameter	0.151645	0.005057
Rosette height	0.243857	0.150933

## Discussion

In this study, we investigated the potential evolutionary mechanisms associated with maintenance of reproductive species barriers between *V. simplex* and *V. scalaris* by examining morphological and genetic variation of these species in the Brazilian Atlantic Forest. Our results revealed three important points: (i) these species can be considered two distinct taxa, supported by genetic and morphological data, even with the occurrence of natural hybridization; (ii) divergent levels of genetic diversity (lower in *V. scalaris*) and *F*_IS_ (higher in *V. scalaris*) are in agreement with reproductive system variation in these species (Neri *et al.* 2017), with predominance of selfing in *V. scalaris* and outcrossing in *V. simplex*; (iii) variation in floral characters among sympatric and allopatric populations occurring only in the outcrosser *V. simplex*, suggests sympatric floral display in *V. simplex* tends to be showier than in allopatry. The variations in reproductive systems and floral traits may be potential prezygotic barriers. Although incomplete, the combination of prezygotic barriers (divergent mating system and floral display) together with postzygotic barriers (inviable hybrid seeds), may act to maintain the morphologic and genetic integrity of these incipient species, even in the presence of hybridization. The different approaches used in this study provide information on the processes involved in maintaining the integrity of correlated species.

### Genetic and morphological differentiation and species integrity

Genetic differentiation between species (AMOVA− *F*_CT_ = 10.99 %, *P*-value < 0.001), differences in distributions of allele frequencies between species (PCoA; Fig. 4) and low levels of interspecific gene flow (*N*_e_*m* = 0.05–0.24; Fig. 5) in sympatric populations of *Vriesea* suggest these species are indeed independent evolutionary units. Despite the differentiation between species supported by genetic and morphologic data, ancestral polymorphisms or recent gene flow (Coyne and Orr 2004) could still be present between these sister species (Barfuss *et al.* 2016). Ancestral polymorphism sharing is likely due to recent species divergence in the *Vriesea* genus (Givnish *et al.* 2014; Gomes-da-Silva 2013; Barfuss *et al.* 2016) and/or incomplete lineage sorting (e.g. Costa *et al.* 2009; Zanella *et al.* 2016). Thus, despite being efficient, these reproductive barriers may still be permeable, with putative hybrids observed in the field and in manual interspecific crosses.

In agreement with genetic data, our CVA clearly indicated morphological discontinuities (Fig. 6), supporting differentiation between the species. Morphological differentiation between *V. simplex* and *V. scalaris* is associated mainly with floral traits (scape length; length and width of the floral bracts; length and width of the bract floral scape; and pistil length) (Table 5). These results suggest that morphological traits between species can be involved in maintaining species boundaries; however, more studies are needed to confirm this hypothesis.

### Hybridization patterns across sympatric populations and reproductive barriers

The STRUCTURE results identified hybrids in all sympatric populations (Fig. 2) confirming previous hypotheses of hybridization between *V. simplex* and *V. scalaris* based on field observations and manual interspecific crosses (Neri *et al.* 2017).

Ours results revealed differences in genetic composition among the sympatric populations studied. The EBS and RUS sympatric populations have higher numbers of hybrids (STRUCTURE analysis) than the RDB sympatric population. In addition, only in EBS and RUS populations were hybrids classified by NEWHYBRIDS, and they were mostly F_2_s, although some hybrids could not be classified (Fig. 3). The ability to identify and classify hybrid individuals through genetic analysis depends ultimately upon the number of diagnostic loci detected (Moccia *et al.* 2007), with different fixed alleles in each species. In this study, most loci were not diagnostic, probably due to the sharing of ancestral polymorphism between these incipient species. Similar results were found in another pair of *Vriesea* species, with difficult to distinguish hybrid classes (Zanella *et al.* 2016).

In this study, hybrid individuals did not present clear morphological distinctions, suggesting that most hybrids may be identified as a parental species based only on morphology (Fig. 6). Although the hybrids are not intermediates, some individuals have an unusual morphology when compared to the parent species. In fact, it is well known that morphological traits alone are limited when identifying natural hybrids, especially considering incipient species (e.g. Li *et al.* 2015a, b; Moreno *et al.* 2015).

Although hybrids with intermediate morphologies were not clearly observed, the occurrence of individuals with intermediate admixture values in all sympatric populations indicates that hybridization events are likely. In agreement with this, there is an overlap of blooming and pollinators (*P*. *eurynome* and *R*. *naevious*) in sympatric areas (Varassin and Sazima 2000; Wendt *et al.* 2008), which potentially favour interspecific pollen exchange. Thus, the overlap of flowering and pollinators can be considered as less effective prezygotic barriers in this system (Wendt *et al.* 2008). However, in the face of hybridization, reproductive isolation may be maintained (Coyne and Orr 2004) and other prezygotic and postzygotic reproductive barriers can contribute to isolation between two species (Rieseberg and Willis 2007; Lowry 2008; Widmer *et al.* 2009).

Our analysis of two hybridizing *Vriesea* species allows us to discuss the general barriers involved in the maintenance of species integrity. Differences in the reproductive system of these species (Neri *et al.* 2017), with the predominance of selfing in *V. scalaris* and outcrossing in *V. simplex*, may be considered as a premating reproductive isolation barrier. We observed sympatric populations in *V. scalaris* with lower *t*_a_ values (0.158–0.358) than in allopatric populations (0.408–0.754). This difference strongly suggests that in sympatry, *V. scalaris* tends to have higher selfing than in allopatry. Furthermore, we observed asymmetric levels of gene flow from *V. scalaris* into *V. simplex* (Fig. 5), suggesting selfing as a potential reproductive barrier between these species. In fact, empirical (Fishman and Wyatt 1999; Martin and Willis 2007; Matallana *et al.* 2010; Brys *et al.* 2013, 2015; Palma-Silva *et al.* 2015) and theoretical (Hu 2015) studies have shown selfing in a potentially interbreeding species can affect rates of interspecific gene flow to an outcrossing species, contributing to reproductive isolation.

Divergent mating systems were reported to contribute as reproductive barriers in other plant species (Fishman and Wyatt 1999; Lowe and Abbott 2004; Fishman and Stratton 2004; Martin and Willis 2007), including bromeliads (Wendt *et al.* 2002; Matallana *et al.* 2010; Palma-Silva *et al.* 2015; Wagner *et al.* 2015). The divergent mating systems and asymmetric levels of gene flow may be a consequence of higher herkogamy (the distance between the stigma and anthers) in *V. simplex* than in *V. scalaris* (Neri *et al.* 2017). Herkogamy in *V. simplex* may increase the possibility of contact with heterospecific pollen. In contrast, in *V. scalaris*, with lower herkogamy, spontaneous selfing can facilitate the protection of stigmas with plant self-pollen, and may counterbalance the input of cross-pollen.

Our morphometric data showed significant variation in floral traits (floral bract length and width, scape bract length and scape length) among sympatric and allopatric populations of *V. simplex* (Fig. 7). Floral bract length and width and scape bract length in *V. simplex* were larger in sympatric populations than in allopatric populations. These results suggest that sympatric floral display in the outcrosser *V. simplex* tends to be showier than in allopatry. In addition, we observed a higher effective migration rate (*N*_e_*m*) towards *V. simplex* in the sympatric populations, which reflects diversity and genetic structure, as well as the variation in flower traits of this species in the hybrid zones.

In contrast, no significant variations in floral traits were found among the sympatric or allopatric populations of *V. scalaris*. In fact, these floral phenotypes are often assumed to be the result of pollinator selection (Barrett and Harder 1996). The divergence of floral traits in sympatric and allopatric populations may evolve in response to competition for pollinator resources, or in response to the costs associated with pollinator sharing between species (Beans *et al.* 2014). Christianini *et al.* (2012) studying sympatric populations of the bromeliad genus *Encholirium* observed that divergence in floral traits and pollinator assemblage may contribute to reproductive isolation between species. Further investigation of the genetic basis of floral traits, including bract colour, in these *Vriesea* species and its interaction with pollination, will shed light on the specific role of floral display in reproductive isolation between closely related species. In *Mimulus* species, two genes altering flower colour were responsible for pollinator shifts and considered an important barrier to maintaining species boundaries (Ramsey *et al.* 2003; Yuan *et al.* 2013). Forthcoming studies on the fitness of sympatric populations versus allopatric populations may indicate whether these floral trait variations are due to reinforcement or to ecological character displacement.

In addition to the prezygotic reproductive barriers discussed above, the low germination rate of interspecific crosses observed in a previous study (Neri *et al.* 2017) suggest that postzygotic isolation may also be involved in maintaining reproductive isolation between *V. simplex* and *V. scalaris*. Lower hybrid seed viability could also explain the low frequency of hybrids observed in nature. Reduced seed viability in interspecific crosses may be due to genetic incompatibility, as in BDM incompatibility (Orr and Turelli 2001; Welch 2004), resulting from negative genetic interaction among nuclear-nuclear loci (Orr and Turelli 2001; Bomblies *et al.* 2007) or cytoplasmic-nuclear loci (Greiner *et al.* 2011). Inviable or sterile hybrids due to genetic incompatibility are potential postzygotic barriers preventing parental species collapse in hybrid zones (Coyne and Orr 2004; Scopece *et al.* 2010). The accumulation of genetic incompatibility was also observed in other pairs of plant species (Moyle and Nakazato 2010; Scopece *et al.* 2008, 2010; Palma-Silva *et al.* 2011; Moyle *et al.* 2012; Ishizaki *et al.* 2013; Briscoe Runquist *et al.* 2014; Johnson *et al.* 2015; Pinheiro *et al.* 2015; Matallana *et al.* 2016).

## Conclusions and Prospects

Here we show that genetic and morphological integrity between *V. simplex* and *V. scalaris* are maintained despite natural hybridization. Our data suggest that in sympatric populations *V. scalaris* tends to have higher selfing rates than in allopatric populations, suggesting that selfing can potentially reduce rates of interspecific gene flow from an outcrossing species. Complementary isolating mechanisms, such as variation in floral traits, among sympatric and allopatric populations in the outcrosser *V. simplex*, may also contribute to the maintenance of species integrity, due to stronger floral display in sympatric populations. The presence of multiple prezygotic and postzygotic barriers and their interactions, although still permeable, probably allow these species to persist in sympatry. While flowering time and pollinator specificities do not appear to be effective prezygotic barriers, we observed that the reproductive system, including floral traits and low seed viability, might contribute to species integrity. To obtain a more complete picture of the species composition of a hybrid zone, it will be necessary in future studies to use a combination of morphological characters and a larger genomic data set that combines nuclear and plastidial markers.

## Sources of Funding

Our work was funded by Fundação de Amparo à Pesquisa do Estado do Rio de Janeiro (FAPERJ E-26/110.944/2013), Fundação de Amparo a Pesquisa do Estado de São Paulo (FAPESP 2009/52725-3), Conselho Nacional de Desenvolvimento Científico e Tecnológico (CNPq – Universal: 490510/2013-2), CNPq/CNR International Cooperation grant (CNPq 490510/2013-2 and 300819/2016-1) and Programa de Pós Graduação em Botânica da Universidade Federal do Rio de Janeiro (Museu Nacional – UFRJ). J.N. received a fellowship from CNPQ-Protax (Nº 52/2010) and CAPES.

## Contributions by the Authors

J.N. conceived the ideas/conducted the collections, conducted experiments, performed the work in the laboratory/analysed data and led the writing of the manuscript. C.P.S. conceived the ideas/conducted the collections/contributed with reagents/materials/analysed data and led the writing of the manuscript. T.W. conceived the ideas/collaborated with materials/revised the writing. This manuscript is part of the PhD thesis of the first author.

## Conflict of Interest

None declared.

## Supporting Information

The following additional information is available in the online version of this article

**Table S1.** Voucher of the populations collected in this study.

**Table S2.** Summary of the morphometrics of *Vriesea simplex*, *V. scalaris* and their hybrids.

## Supplementary Material

Supporting_Information
