# Supplementary material for: Natural hybridization and genetic and morphological variation between two epiphytic bromeliads
Source: AoB Plants. 2017 Nov 25;10(1):plx061. doi: 10.1093/aobpla/plx061 (PMC5751037; doi:10.1093/aobpla/plx061)
Supplement: Supporting_Information [file plx061_suppl_supporting_information.doc]

**Supporting information**

**Table S1. Voucher of collected populations present in this study**

| **Species** | **Populations** | **Code** | **State** | **City** | **Voucher** | **Herbarium** |
| --- | --- | --- | --- | --- | --- | --- |
| *V. scalaris* | Vale do Capão | VAC | Bahia | Palmeiras | J.Neri 191 | MBML |
| *V. scalaris* | Sincorá | SIN | Bahia | Igatú | J.Neri 197 | MBML |
| *V. simplex* | Duas Bocas | RDB | Espírito Santo | Cariacica | J.Neri 132 | MBML |
| *V. scalaris* | Duas Bocas | RDB | Espírito Santo | Cariacica | J.Neri 131 | MBML |
| *V. simplex* | Santa Lúcia | EBS | Espírito Santo | Santa Teresa | J. Neri, Santos M 231 | MBML |
| *V. scalaris* | Santa Lúcia | EBS | Espírito Santo | Santa Teresa | J.Neri, Santos M, 208 | MBML |
| *V. simplex* | Guapimirim | GUA | Rio de Janeiro | Guapimirim | J. Neri et al 105 | R |
| *V. scalaris* | Tijuca | TIJ | Rio de Janeiro | Rio de Janeiro | J.Neri, Perez F 121 | MBML |
| *V. scalaris* | Paquequer | PAQ | Rio de Janeiro | Teresópolis | J.Neri, 125 | MBML |
| *V. simplex* | Soberbo | SOB | Rio de janeiro | Teresópolis | J.Neri, Gonçalves F 141 | MBML |
| *V. simplex* | Santa Virgínia | SVI | São Paulo | São Luiz do Paraitinga | J.Neri, Neves B 147 | MBML |
| *V. scalaris* | Peri | PER | Santa Catarina | Florianópolis | J.Neri 114 | MBML |

**Table S2:** Summary of morphometric of *Vriesea simplex, V. scalaris* and Hybrids*.* Means ± SE (standard error). The *F*-values of ANOVA and *P* are shown. (P<0.05).

| Variables/ taxa | *V. simplex* (N=59) | *V. scalaris* (N=76) | Hybrids (N=4) | F | *P* |
| --- | --- | --- | --- | --- | --- |
| Leafs heath Width | 5.74±0.08 | 5.35±0.49 | 4.77±0.26 | 0.320 | 0.727 |
| Leafs heath length | 7.87±0.14 | 6.58±0.10 | 6.00±0.20 | 29.47 | 0.000 |
| Leaf blade width | 2.90±0.07 | 2.31±0.04 | 2.25±0.13 | 27.19 | 0.000 |
| Leaf blade length | 20.64±0.62 | 15.06±0.46 | 15.82±2.06 | 27.20 | 0.000 |
| Inflorescence total length | 54.65±2.21 | 31.34±0.94 | 34.2±4.49 | 57.75 | 0.000 |
| Scape length | 32.60±1.09 | 15.91±0.50 | 19.0±3.02 | 114.46 | 0.000 |
| Rachis length | 19.77±1.17 | 13.05±0.55 | 13.12±2.86 | 16.23 | 0.000 |
| Flowers number | 8.82±0.36 | 6.21±0.23 | 6.5±1.65 | 19.25 | 0.000 |
| Floral bract length | 4.34±0.005 | 3.23±0.003 | 3.45±0.21 | 149.6 | 0.000 |
| Floral bract width | 2.69±0.06 | 1.86±0.003 | 2.07±0.18 | 82.25 | 0.000 |
| Sepal length | 3.56±0.004 | 3.27±0.004 | 3.30±0.10 | 10.43 | 0.000 |
| Sepal width | 1.36±0.004 | 1.26±0.004 | 1.22±0.02 | 1.323 | 0.270 |
| Petal length | 4.46±0.004 | 3.62±0.006 | 4.02±0.13 | 49.52 | 0.000 |
| Petal width | 0.56±0.014 | 0.55±0.056 | 0.42±0.02 | 0.267 | 0.766 |
| Scape bracts length | 4.05±0.061 | 3.04±0.036 | 3.32±0.31 | 107.8 | 0.000 |
| Scape bracts width | 2.39±0.054 | 1.58±0.04 | 1.82±0.30 | 63.26 | 0.000 |
| Anther length | 0.58±0.011 | 0.43±0.05 | 0.45±0.05 | 3.095 | 0.048 |
| Lenth pistil | 5.52±0.09 | 4.12±0.06 | 4.70±0.31 | 76.69 | 0.000 |
| Pedicle length | 1.67±0.04 | 1.32±0.030 | 1.65±0.15 | 23.33 | 0.000 |
| Anther-stigma distance | 0.75±0.06 | 0.28±0.05 | 0.12±0.07 | 19.07 | 0.000 |
| Stamen length | 4.95±0.06 | 3.99±0.06 | 4.67±0.32 | 59.10 | 0.000 |
| Filite length | 4.35±0.06 | 3.65±0.04 | 4.22±0.27 | 46.6 | 0.000 |
| Rosette diameter | 35.43±1.06 | 28.56±0.78 | 32.00±4.43 | 14.03 | 0.000 |
| Rosette height | 30.23±0.62 | 22.77±0.51 | 22.87±2.67 | 42.85 | 0.000 |
